# Associations of *Anaplasma phagocytophilum* Bacteria Variants in *Ixodes scapularis* Ticks and Humans, New York, USA

**DOI:** 10.3201/eid2903.220320

**Published:** 2023-03

**Authors:** Melissa Prusinski, Collin O’Connor, Alexis Russell, Jamie Sommer, Jennifer White, Lauren Rose, Richard Falco, John Kokas, Vanessa Vinci, Wayne Gall, Keith Tober, Jamie Haight, JoAnne Oliver, Lisa Meehan, Lee Ann Sporn, Dustin Brisson, P. Bryon Backenson

**Affiliations:** New York State Department of Health, Albany, New York, USA (M. Prusinski, A. Russell, J. Sommer, J. White, L. Rose, L. Meehan, P.B. Backenson);; University at Buffalo Department of Geography, Buffalo, New York, USA (C. O’Connor);; New York State Department of Health, Buffalo (C. O’Connor, W. Gall, K. Tober);; New York State Department of Health, Armonk, New York, USA (R. Falco, J. Kokas, V. Vinci);; New York State Department of Health, Falconer, New York, USA (J. Haight);; New York State Department of Health, Syracuse, New York, USA (J. Oliver);; Paul Smith's College, Paul Smiths, New York, USA (L.A. Sporn);; University of Pennsylvania, Philadelphia, Pennsylvania, USA (D. Brisson)

**Keywords:** anaplasmosis, *Anaplasma phagocytophilum*, bacteria, zoonoses, vector-borne infections, *Ixodes*, ticks, spatiotemporal analysis, space-time clustering, geographic information systems, population genetics, genetic variants, public health surveillance, New York, United States, *Suggested citation for this article*: Prusinski M, O’Connor C, Russell A, Sommer J, White J, Rose L, et al. Associations of *Anaplasma phagocytophilum* bacteria variants in *Ixodes scapularis* ticks and humans, New York, USA. Emerg Infect Dis. 2023 Mar [*date cited*]. https://doi.org/10.3201/eid2903.220320

## Abstract

Anaplasmosis, caused by the tickborne bacterium *Anaplasma phagocytophilum*, is an emerging public health threat in the United States. In the northeastern United States, the blacklegged tick (*Ixodes scapularis*) transmits the human pathogenic genetic variant of *A. phagocytophilum* (Ap-ha) and a nonpathogenic variant (Ap-V1). New York has recently experienced a rapid and geographically focused increase in cases of anaplasmosis. We analyzed *A. phagocytophilum–*infected *I. scapularis* ticks collected across New York during 2008–2020 to differentiate between variants and calculate an entomological risk index (ERI) for each. Ap-ha ERI varied between regions and increased in all regions during the final years of the study. Space-time scan analyses detected expanding clusters of Ap-ha located within documented anaplasmosis hotspots. Ap-ha ERI was more positively correlated with anaplasmosis incidence than non-genotyped *A. phagocytophilum* ERI. Our findings help elucidate the relationship between the spatial ecology of *A. phagocytophilum* variants and anaplasmosis.

*Anaplasma phagocytophilum* is the bacterium capable of causing anaplasmosis (*1*,*2*). Anaplasmosis symptoms include fever, headache, myalgia, and malaise (*3*). Severe illness is reported more commonly in older and immunocompromised patients but is also documented in immunocompetent persons and may result in hospitalization or death if appropriate treatment is not promptly administered (*3*). Patient condition generally improves markedly in the 24–48 hours after initiation of treatment with the antimicrobial drug doxycycline (*3*).

In eastern North America, *A. phagocytophilum* is transmitted to humans through the bite of an infected blacklegged tick (*Ixodes scapularis*) (*2*). *I. scapularis* ticks have 3 active life stages (larva, nymph, and adult); nymphs and adults can carry multiple genetic variants of *A. phagocytophilum*, including the pathogenic human-active variant (Ap-ha) and the nonpathogenic Variant 1 (Ap-V1) (*4*–*6*). Other genetic variants of *A. phagocytophilum* have been documented in the northeastern United States; their prevalence in nature and pathogenicity remains understudied (*7*,*8*). *A. phagocytophilum* is not transmitted transovarially from infected adult female *I. scapularis* ticks to larvae and is maintained in the environment within various host species (*9*,*10*). The most common host reservoir of Ap-ha is the white-footed mouse (*Peromyscus leucopus*) and of Ap-V1 is the white-tailed deer (*Odocoileus virginianus*) (*5*,*8*,*11*).

In New York state, excluding the city of New York, 5,146 anaplasmosis cases were reported during 2010–2018; the median was 454 (range 220–1,112) cases/year. Anaplasmosis incidence increased statewide nearly 4-fold over a period of a decade, from 2.0 cases/100,000 persons in 2010 to 7.6 cases/100,000 persons during 2018; that increase was not spatially homogenous (*12*,*13*). Specifically, the largest increase in anaplasmosis incidence occurred in the Capital District region of New York, where incidence increased from 3.0/100,000 persons in 2008 to 5.3/100,000 persons in 2018 (*13*). Focal increases in incidence of anaplasmosis may result from a change in the abundance and spatial extent of Ap-ha–infected *I. scapularis* ticks, potentially related to changes in the deer–tick–rodent cycle (*10*); those changes may reflect the relative abundance of Ap-ha competent reservoir hosts, increased contact between host-seeking ticks (unfed ticks of any lifestage actively seeking a host bloodmeal) and small mammal reservoirs of Ap-ha, or a greater overlap between human residential or recreational areas and habitats conducive for the enzootic amplification of Ap-ha. Thus, further examination of the relationship between Ap-ha and Ap-V1 may broaden the understanding of anaplasmosis etiology and *A. phagocytophilum* ecology, refining risk assessment for this emerging disease and enabling targeted prevention efforts to reduce anaplasmosis incidence.

We analyzed *A. phagocytophilum*–infected host-seeking *I. scapularis* tick specimens to elucidate the spatial differences in entomological risk for anaplasmosis in New York. We used a TaqMan single-nucleotide polymorphism (SNP) genotyping assay (ThermoFisher Scientific, https://www.thermofisher.com) to differentiate between the Ap-ha and Ap-V1 variants as previously described (*14*). Next, we calculated a measure of human-infection risk as a function of *I. scapularis* tick density and *A. phagotycophilum* genotype prevalence, known as the entomological risk index (ERI) for both Ap-ha and Ap-V1.We then tested for spatiotemporal differences in counts of Ap-ha– and Ap-V1–infected *I. scapularis* ticks by using statistical modeling and tested for correlations between Ap-ha ERI and anaplasmosis incidence. We also used a scan statistic to search for spatiotemporal clusters of Ap-ha and Ap-V1 in New York tick populations for 2008–2020. We then compared spatiotemporal clusters of Ap-ha and Ap-V1 in *I. scapularis* ticks to documented regions of increased anaplasmosis incidence in New York over the same timeframe (*13*,*15*). The results help to illuminate the relationship between the spatial ecology of each variant and the outcome of human disease.

## Methods

### Active Tick Sampling

**Figure 1:**
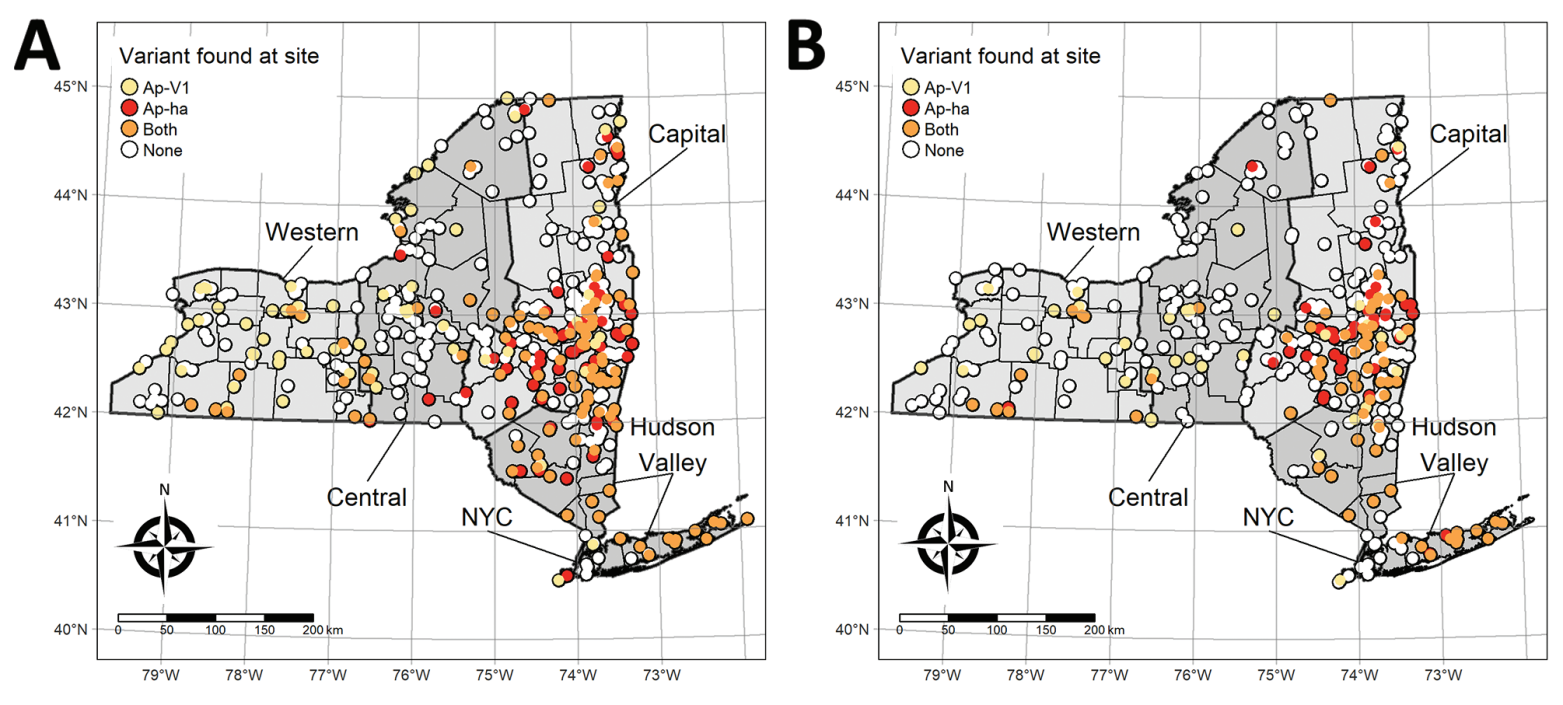
New York State Department of Health (NYSDOH) *Ixodes scapularis* tick sampling sites categorized by the *Anaplasma phagocytophilum* genetic variants found at each site, New York, USA. Thick black outlines indicate NYSDOH regions (labeled). A) Adult sampling sites; B) nymph sampling sites*.*

We collected host-seeking ticks primarily from publicly accessible lands across New York during 2008–2020 using standardized drag-cloth or flag surveys of vegetation and forest leaf litter, as previously described (*16*). Generally, we visited 1 site within each county twice annually; we visited additional sites as weather and resources permitted. Collection sites had suitable tick habitat and potential risk for human exposure to ticks. We typically sampled >1,000 m^2^ of suitable habitat per site during each collection event. In some instances, we did not find suitable habitat for nymphal and adult *I. scapularis* tick sampling at the same collection site; as a result, we sampled some sites for 1 *I. scapularis* lifestage, resulting in separate nymphal and adult *I. scapularis* tick sampling sites (Figure 1). We stored ticks in 70%–100% ethanol at 4°C until they were sorted by developmental stage and identified to species using dichotomous keys (*17*,*18*), placed into sterile microcentrifuge tubes containing 100% ethanol, and stored at −20°C until DNA extraction (*16*,*19*).

### Pathogen Detection and Ap-ha/Ap-V1 Differentiation

Individual *I. scapularis* nymphs and adults underwent total genomic DNA extraction as previously described (*16*,*19*). Using a quadplex real-time PCR (*20*), we tested for the following pathogens and target genes: *A. phagocytophilum* (msp 2), *Babesia microti* (18S rDNA), *Borrelia burgdorferi* (16S rDNA), and *Borrelia miyamotoi* (16S rDNA). Samples testing positive for *A. phagocytophilum* by quadplex PCR were further tested using a custom TaqMan SNP genotyping PCR to differentiate between the Ap-ha and Ap-V1 variants of *A. phagocytophilum* as described previously (*14*), with the following modifications: each 25 uL reaction contained 0.625 uL of 80× Custom TaqMan SNP Genotyping Assay primer/probe mix, 12.5 uL TaqMan Universal Master Mix (ThermoFisher), 1.875 uL nuclease-free water, and 10 uL of gDNA template (or nuclease-free water for negative controls). We performed SNP assays and variant assignment and generated allelic discrimination plots using Applied Biosystems 7500 Real-time PCR System version 2.0.5 (ThermoFisher). 

### ERI Calculation

**Figure 6:**
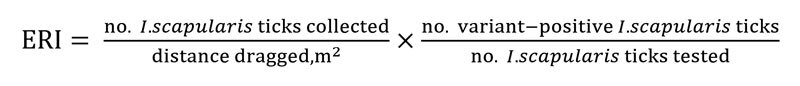
Equation calculating ERI for *I. scapularis* nymphs and adults.

To estimate risk for human exposure to Ap-ha and Ap-V1 variants of *A. phagocytophilum*, we calculated ERI (*20*) using the equation (Figure 6) for *I. scapularis* nymphs and adults. A high ERI value denotes an increased risk for human exposure to a particular pathogen. Of note, Ap-V1 does not cause disease in humans, so the term entomologic risk index is used only to provide a metric for comparison between Ap-ha and Ap-V1 variants. For sites with multiple sampling events within the same season, we averaged ERIs for all events.

### Bernoulli Space-Time Scan Statistic

We compared the spatiotemporal distribution of Ap-ha–infected and Ap-V1–infected *I. scapularis* adults and nymphs by using the Bernoulli space-time scan statistic (*21*), implemented in SaTScan version 9.6 (https://www.satscan.org) (*22**,**23*). SaTScan searched for statistically significant clusters of Ap-ha or Ap-V1; we considered a cluster to be a location at which the relative risk (RR) of the presence of a variant is >1.0 inside a given cluster compared to outside. We selected maximum spatial and temporal cluster sizes a priori for our analysis. We set maximum spatial cluster size as 10% of the collected ticks to allow for new clusters to form as time progressed and to show the movement of each variant. We selected maximum temporal cluster size as 90% of the study period to allow for the identification of established populations of either *A. phagocytophilum* variant.

### Statistical Analysis

We tested the Spearman rank correlation between mean Ap-ha ERI in *I. scapularis* nymphs and adults and anaplasmosis incidence at the postal (ZIP) code tabulation area level gathered from the New York State Department of Health (NYSDOH) Communicable Disease Electronic Surveillance system as previously described (*13*). We assessed the correlation for each year, 2010–2018. We selected the Spearman rank test because of the underlying count data used to generate anaplasmosis incidence and Ap-ha ERI. We corrected the 18 correlation tests for multiple testing using the Bonferroni-Holm adjustment (*24*). We compared results of the Spearman rank tests with the results from a previous analysis using non–genotype-specific *A. phagocytophilum* ERI (*13*).

We tested for spatiotemporal interaction in the number of Ap-ha– and Ap-V1–infected *I. scapularis* nymphs and adults by year and across latitude and longitude categories using a generalized linear mixed model (GLMM) extension of zero-inflated negative binomial (ZINB) regression (*25**,**26*). ZINB regression accounted for overdispersion and excess zero-counts of Ap-ha–and Ap-V1–infected *I. scapularis* ticks, whereas the GLMM extension handled the repeated nature of our sampling scheme by allowing sampling sites to have varying intercepts. We binned tick collection data and corresponding PCR results by year, latitude, and longitude to increase the number of observations within each combination of covariates to fit the model. We binned tick data by year into 4 categories: 2008–2011, 2012–2014, 2015–2017, and 2018–2020. We binned tick collection sites by latitude into 3 categories: sites south of 42°N, at 42°N to 43°N, and north of 43°N. We binned collection sites by longitude into 3 categories: sites east of 74°W, from 74°W to 76°W, and west of 78°W. We built 4 models to analyze Ap-ha and Ap-V1 in *I. scapularis* nymphs and adults separately. We assessed interaction between year and latitude/longitude categories using the likelihood ratio test (LRT). We used the natural log of the total number of *I. scapularis* ticks of the target developmental stage (nymphs during sampling events in late May, June, July, and August; adult ticks during April, early May, October, November, and December) as an offset. We conducted data cleaning, generation of summary statistics, and data visualization using R version 4.0.3 (http://www.rstudio.com) and the dplyr (https://CRAN.R-project.org/package=dplyr), sf, ggplot2, and tmap R packages (*27*–*29*). We used the glmmTMB package in R (*30*) for modeling.

## Results

### Active Tick Sampling and Pathogen Detection

We recorded active tick sampling and *A. phagocytophilum* genotyping results for *I. scapularis* tick specimens (Table 1). We categorized sampling sites according to the presence of *A. phagocytophilum* genetic variants (Figure 1). Of 91,163 adult *I. scapularis* ticks collected during 2008–2020, we tested 43,520 for *A. phagocytophilum*; of 38,782 nymphal ticks, we tested 25,748 (Table 1). Among those tested for *A. phagocytophilum*, 3,207 (7.4%) adults and 1,183 (4.6%) nymphs were determined to be positive. *A. phagocytophilum* genotyping deterimined that *I. scapularis* adults had a higher prevalence of Ap-ha (5.4%) than Ap-V1 (1.7%), whereas *I. scapularis* nymphs had a higher prevalence of Ap-V1 (2.6%) than Ap-ha (1.7%). We observed co-infection of Ap-ha and Ap-V1 in *I. scapularis* adults (0.04%).

**Table 1:** Sampling and genotyping analysis results of adult and nymphal *Ixodes scapularis* ticks collected in New York, USA, 2008–2020*

Characteristic	Adults	Nymphs
No. site visits	2,496	1,595
No. specimens collected	91,163	38,782
Specimens tested, no. (%)	43,520 (47.74)	25,748 (66.39)
*A. phagocytophilum* positive	3,207 (7.37)	1,183 (4.59)
Specimens genotyped, no. (%)		
Ap-ha positive	2,327 (5.35)	425 (1.65)
Ap-V1 positive	728 (1.67)	669 (2.60)
Ap-ha/Ap-V1 co-infected	18 (0.04)	0 (0.00)
Undetermined	124 (0.28)	87 (0.34)
Missing†	10 (0.02)	2 (0.01)

*Ap-ha, human pathogenic variant of A. phagocytophilum bacteria; Ap-V1, nonpathogenic variant of *A. phagocytophilum* bacteria. †Refers to samples that were unable to be located in storage for genotyping.

### ERI

**Figure 2:**
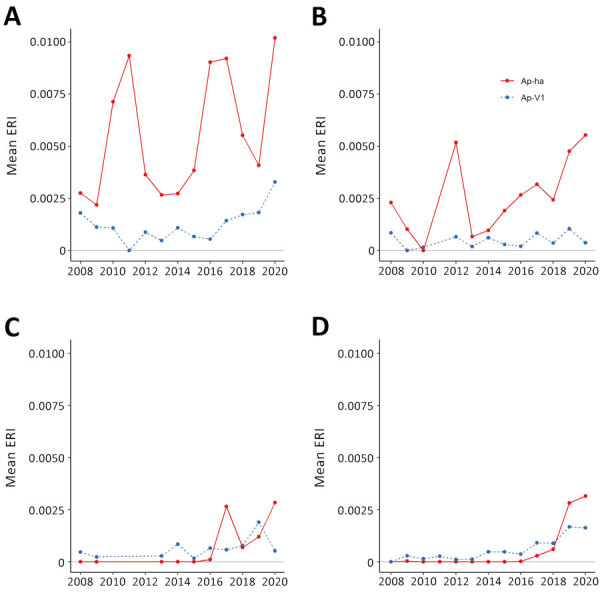
Mean ERI of pathogenic and nonpathogenic genetic variants of *Anaplasma phagocytophilum* bacteria in adult blacklegged ticks aggregated to regions of New York, 2008–2020. A) Hudson Valley region; B) Capital region; C) Central region; D) Western region. Ap-ha, human pathogenic variant of *A. phagocytophilum* bacteria; Ap-V1, nonpathogenic variant of *A. phagocytophilum*; ERI, entomological risk index.

**Figure 3:**
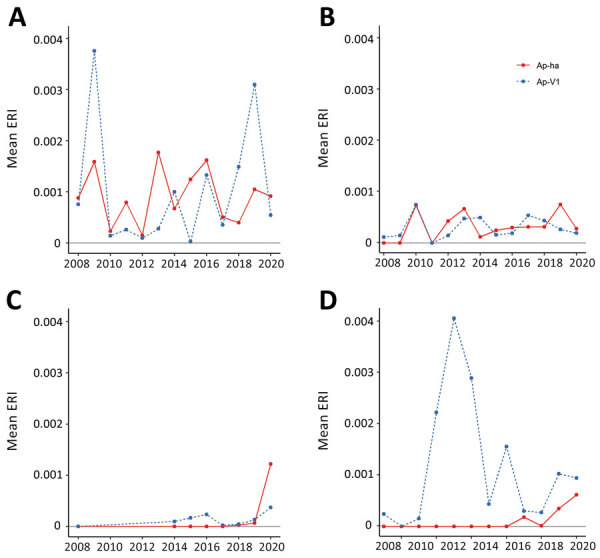
Mean ERI of pathogenic and nonpathogenic genetic variants of *Anaplasma phagocytophilum* bacteria in nymphal blacklegged ticks aggregated to regions of New York, 2008–2020. A) Hudson Valley region; B) Capital region; C) Central region; D) Western region. Ap-ha, human pathogenic variant of *A. phagocytophilum* bacteria; Ap-V1, nonpathogenic variant of *A. phagocytophilum;* ERI, entomological risk index.

The Hudson Valley and Capital District regions in the eastern portion of the state generally exhibited higher Ap-ha ERI than the Central and Western regions of New York (Figures 2, 3). Ap-V1 ERI of *I. scapularis* nymphs was generally higher in the Western and Hudson Valley regions than in the Capital District and Central NY regions, but the levels were highly variable and exhibited no obvious temporal trend. Overall, ERI for Ap-ha increased in the later years of the study period for all 4 regions.

### Retrospective Bernoulli Space-Time Cluster Analysis

**Figure 4:**
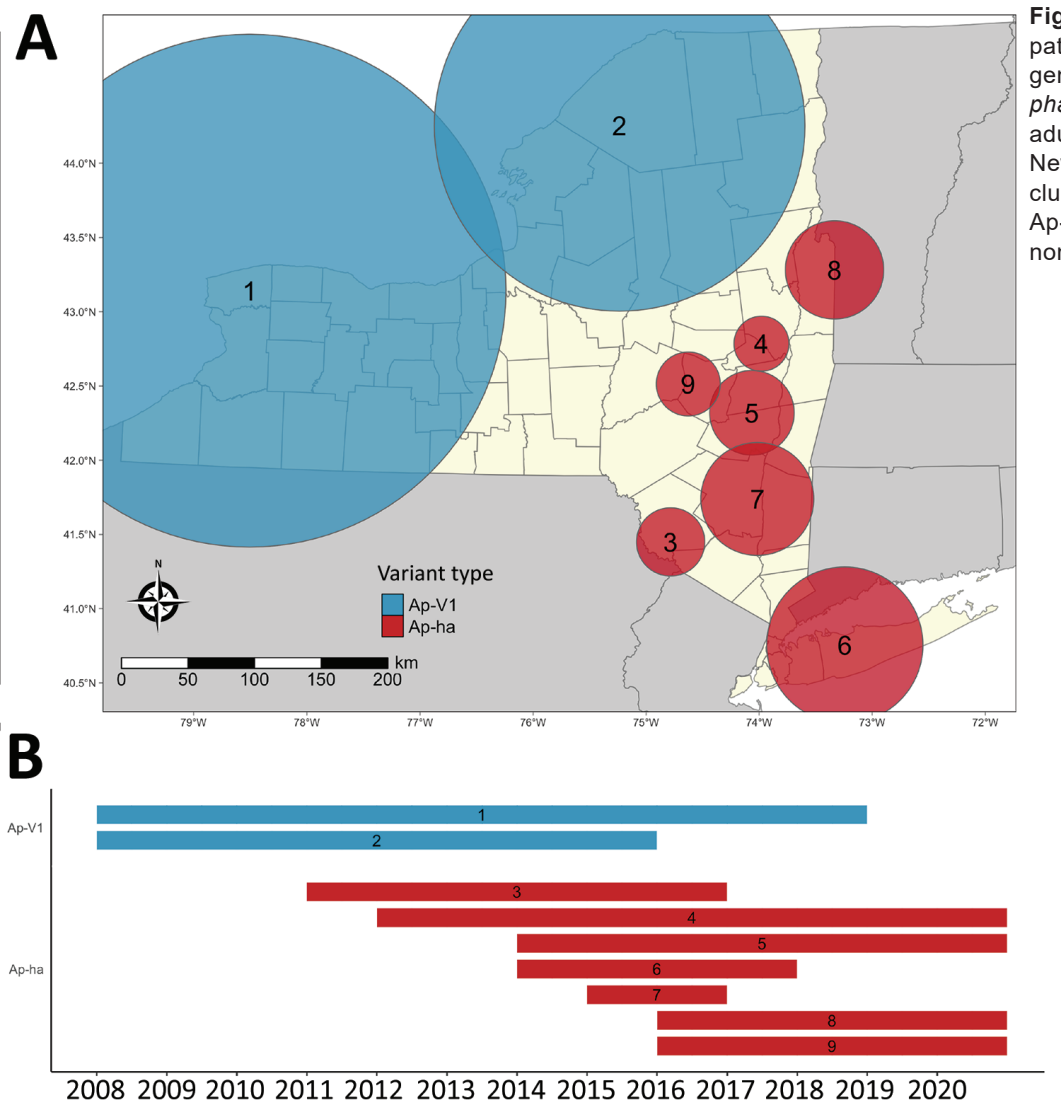
Bernoulli clusters of pathogenic and nonpathogenic genetic variants of *Anaplasma phagocytophilum* bacteria in adult *Ixodes scapularis* ticks in New York, 2008–2020. A) Spatial clusters; B) temporal clusters. Ap-ha, pathogenic variant; Ap-V1, nonpathogenic variant.

**Figure 5:**
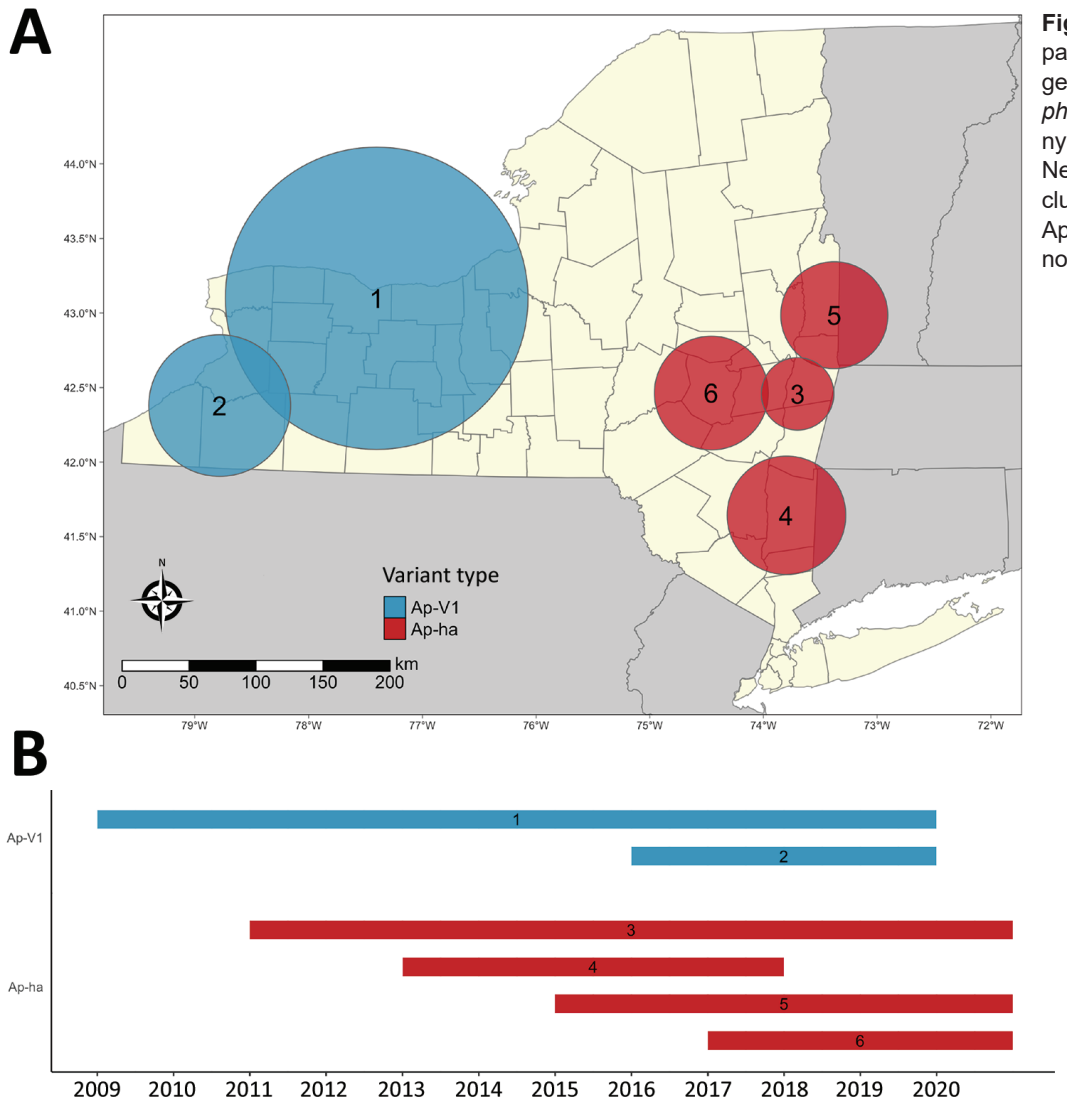
Bernoulli clusters of pathogenic and nonpathogenic genetic variants of *Anaplasma phagocytophilum* bacteria in nymphal *Ixodes scapularis* ticks in New York, 2008–2020. A) Spatial clusters; B) temporal clusters. Ap-ha, pathogenic variant; Ap-V1, nonpathogenic variant.

We detected increased RR of Ap-ha or Ap-V1 in 9 clusters of adult (Table 2; Figure 4, panel A) and 6 clusters of nymphal *I. scapularis* (Figure 5, panel A) ticks in the period 2008–2020. Among the 9 clusters of *I. scapularis* adults, 7 exhibited increased RR of Ap-ha and 2 exhibited increased RR of Ap-V1. Among the 6 clusters of *I. scapularis* nymphs, 4 exhibited increased RR of Ap-ha and 2 exhibited increased RR Ap-V1. Clusters of Ap-ha tended to be located in the Hudson Valley and eastern Capital District regions of New York, whereas clusters of Ap-V1 tended to be located in the Western and northern Capital District regions of New York, near the border with Canada. Analysis of *I. scapularis* adults revealed 3 of 13 years in our study period with no clusters of Ap-ha (2008–2010), whereas clusters of Ap-V1 were present in all years but 2019 and 2020 (Figure 4). Analysis of *I. scapularis* nymphs exhibited similar results; clusters of Ap-ha were present in all but 2 years of the study period (2009 and 2010), and 1 year, 2020, was without a cluster of Ap-V1 (Figure 5).

**Table 2:** Distance and relative risks of space-time Bernoulli clusters of pathogenic and nonpathogenic variants of *A. phagocytophilum* bacteria in adult and nymphal *Ixodes scapularis* ticks, New York, USA, 2008–2020*

Cluster no.	Cluster type	Years	Radius, km	RR
Adults				
1	Ap-V1	2008–2018	192.47	4.74†
2	Ap-V1	2008–2015	139.14	4.23†
3	Ap-ha	2011–2016	25.62	1.29
4	Ap-ha	2012–2020	20.64	1.22
5	Ap-ha	2014–2020	31.76	1.26
6	Ap-ha	2014–2017	58.66	1.24
7	Ap-ha	2015–2016	42.41	1.30
8	Ap-ha	2016–2020	36.91	1.28
9	Ap-ha	2016–2020	24.01	1.24

Nymphs				
1	Ap-V1	2009–2019	113.01	1.80†
2	Ap-V1	2016–2019	52.94	1.60†
3	Ap-ha	2011–2020	27.10	1.66
4	Ap-ha	2013–2017	44.28	2.12
5	Ap-ha	2015–2020	39.95	2.02
6	Ap-ha	2017–2020	42.53	2.19

*Ap-ha, human pathogenic variant of *A. phagocytophilum* bacteria; Ap-V1, nonpathogenic variant of *A. phagocytophilum* bacteria; RR, relative risk. †Case and control groups were reversed to indicate an increased risk of one variant to another.

### Spearman Rank Correlations and Zero-Inflated Regression Models

Anaplasmosis incidence was significantly correlated with Ap-ha ERI in *I. scapularis* adults for all 9 years analyzed, whereas 6 of the 9 years analyzed were correlated for *I. scapularis* nymphs (Table 3). Statistically significant correlation coefficients in *I. scapularis* adults were 0.36–0.75, an increase in the minimum and maximum correlation coefficients compared with non–variant-specific PCR results (*13*). Statistically significant correlation coefficients in *I. scapularis* nymphs were 0.19–0.68, a decrease in the minimum coefficient and an increase in the maximum coefficient compared with correlations calculated using the non–variant-specific PCR results (*13*).

**Table 3:** Spearman correlation results between Ap-ha ERI and anaplasmosis incidence at postal code tabulation area level, New York, USA, 2010–2018*

Year	Adults			Nymphs	
	ρ	p value		ρ	p value
2010	0.75	0.0011†		0.24	0.3591
2011	0.75	0.0185†		−0.21	0.5524
2012	0.62	0.0001†		0.57	0.0233†
2013	0.52	<0.0001†		0.68	0.0001†
2014	0.57	<0.0001†		0.20	0.1225
2015	0.73	<0.0001†		0.56	<0.0001†
2016	0.71	<0.0001†		0.43	0.0001†
2017	0.57	<0.0001†		0.47	0.0001†
2018	0.36	<0.0001†		0.19	0.0407†

*Ap-ha, human pathogenic variant of *A. phagocytophilum* bacteria; ERI, entomological risk index; ρ, Spearman rank correlation coefficient;. †p<0.05, indicating statistical significance.

ZINB regression models of Ap-ha and Ap-V1 in *I. scapularis* nymphs failed to converge, likely because of an insufficient number of observations within the year, latitude, and longitude covariate combinations. We detected notable spatiotemporal interaction only in Ap-ha in *I. scapularis* adults (Table 4). The final model for Ap-ha–infected *I. scapularis* adults indicated high interaction between both year and latitude and year and longitude in the negative binomial portion of the model; however, the LRT indicated the model with year and longitude interaction was not better fit than the model without the year and longitude interaction (p = 0.0976). (Table 4). Model coefficients indicate increasing log counts of Ap-ha–infected *I. scapularis* adults north of 43°N as year categories increased: for 2012–2014, β = 2.36 (95% CI 0.32–4.40); 2015–2017, β = 2.76 (95% CI 0.76–4.47); and 2018–2020, β = 3.79 (95% CI 1.77–5.80). log counts of Ap-ha–infected *I. scapularis* adult at latitudes from 42°N to 43°N only increased in the final year category: for 2012–2014, β = 0.27 (95% CI −0.60 to 1.14); 2015–2017, β = 0.50 (95% CI −0.34 to 1.33); and 2018–2020, β = 1.39 (95% CI 0.56– 2.21). In addition, the log counts increased in year category 2018–2020 between 74°W and 76°W (β = 0.78 [95% CI 0.02–1.53]), although the LRT indicated that using the year and longitude interaction did not improve model fit. Of note, the interaction between the 2012–2014 year category and west of 76°W longitude category exhibited a wide 95% CI (−6,096.27 to 6,062.91) because no positive Ap-ha *I. scapularis* ticks were found in all 68 site visits at those latitudes over that period. The zero-inflated portion of the model indicated a significant difference in the log odds of being an excessive zero between all latitude categories and longitude categories. For latitudes between 42°N and 43°N, β = 2.15 (95% CI 0.31–3.99); north of 43°N, β = 3.82 (95% CI 1.92–5.72). For longitudes between 74°W and 76°W, β = 1.30 (95% CI 0.53– 2.08); west of 76°W, β = 3.95 (95% CI 2.96–4.94). Only the 2018–2020 year category significantly differed from the reference group: for that category, β = −1.68 (95% CI −3.31 to −0.06).

**Table 4:** Final zero-inflated negative binomial regression model for Ap-ha in adult *I. scapularis* in New York, USA, 2010–2018

Category	Negative binomial model					Zero-inflated model			
	β	95% CI	SE	p value		β	95% CI	SE	p value
Intercept	–3.19	–3.69 to –2.69	0.25	**<0.0001**		–3.67	–6.14 to –1.20	1.26	**0.0036**
Year categories									
2008–2011	Referent					Referent			
2012–2014	–0.09	–0.65 to 0.46	0.29	0.7431		–0.18	–1.83 to 1.47	0.84	0.8282
2015–2017	0.33	–0.18 to 0.83	0.26	0.2023		–0.23	–1.80 to 1.35	0.80	0.7783
2018–2020	–0.40	–0.90 to 0.10	0.26	0.1149		–1.68	–3.31 to –0.06	0.83	**0.0421**
Latitude categories									
South of 42°N	Referent					Referent			
42°N–43°N	–0.50	–1.31 to 0.32	0.42	0.2304		2.15	0.31–3.99	0.94	**0.0223**
North of 43°N	–2.91	–4.93 to –0.89	1.03	**0.0048**		3.82	1.92–5.72	0.97	**<0.0001**
Longitude categories									
East of 74°W	Referent								
74°W–76°W	–0.63	–1.39 to 0.14	0.39	0.1071		1.30	0.53–2.08	0.40	**0.0001**
West of 76°W	–1.67	–4.44 to 1.09	1.41	0.2351		3.95	2.96–4.94	0.50	**<0.0001**
Interaction categories									
2012–2014: 42°N–43°N	0.27	–0.60 to 1.14	0.44	0.5456					
2015–2017: 42°N–43°N	0.50	–0.34 to 1.33	0.43	0.2435					
2018–2020: 42°N–43°N	1.39	0.56–2.21	0.42	**0.0010**					
2008–2011: North of 43°N)	Referent								
2012–2014: North of 43°N	2.36	0.32–4.40	1.04	**0.0237**					
2015–2017: North of 43°N	2.76	0.76–4.47	1.02	**0.0069**					
2018–2020: North of 43°N	3.79	1.77–5.80	1.03	**0.0002**					
2012–2014: 74°W–76°W	0.45	–0.38 to 1.27	0.42	0.2915					
2015–2017: 74°W–76°W	0.33	–0.43 to 1.09	0.39	0.3948					
2018–2020: 74°W–76°W	0.78	0.02–1.53	0.38	**0.0437**					
2012–2014: West of 76°W	–16.73	–6,096.37 to 6,062.91	3,101.91	0.9957					
2015–2017: West of 76°W	0.77	–2.06 to 3.60	1.44	0.5917					
2018–2020: West of 76°W	1.25	–1.54 to 4.04	1.42	0.3799					

*Bold indicates statistical significance. Ap-ha, human pathogenic variant of *A. phagocytophilum* bacteria; ERI, entomological risk index.

## Discussion

Our study describes the landscape of Ap-ha and Ap-V1 genetic variants in New York, which has direct public health implications on the incidence of anaplasmosis. Continued geographic expansion of the Ap-ha variant in New York, as shown in this study, will result in a growing area of increased anaplasmosis risk for residents of the impacted regions. The current distribution of *A. phagocytophilum* variants and associated anaplasmosis risk in New York is characterized by elevated risk in the Hudson Valley and Capital District region predominated by Ap-ha, compared with other geographic regions with low or variable Ap-V1 prevalence (Figure 2). During our study, the range of Ap-ha expanded relative to Ap-V1 over time, whereas Ap-V1 was largely unchanged and remained the dominant variant in the Western and northern Capital District regions. Those regions border the Canada provinces Ontario and Quebec, where Krakowetz et al. (*14*) also found Ap-V1 to be the predominant variant, indicating the range of Ap-V1 may extend from the spatial clusters detected in our analysis northward into both provinces.

The results from the ZINB regression model support our variant cluster detection; Ap-ha expanded northward at an increasing rate over time, and some westward expansion is evident. Of note, these directions are relative only to the spatial extent of New York; our analyses only examined New York data. Including data from Massachusetts, Connecticut, and Vermont could indicate a westward expansion of entomological risk from Ap-ha. If the true spread of Ap-ha is occurring radially from neighboring states east of New York, the phenomenon would appear as the northward and westward increase relative to the borders of New York, as we observed in our results.

The geographic dynamics of Ap-ha and Ap-V1 are also likely linked to the deer–tick–rodent cycle and the varied reservoir competency of key vertebrate hosts. The observed geographic range expansion of Ap-ha, whereas that of Ap-V1 remained stable, indicates that the variants may not have a directly inverse relationship. Furthermore, co-infection of Ap-ha and Ap-V1 within individual ticks was rarely observed in our study and others (*14*), suggesting some competitive interaction between pathogen variants within the vector. The varied *A. phagocytophilum* genotype prevalence across *I. scapularis* tick life stages points to a developmental stage-specific association; the exact ecologic mechanism is unknown. One possibility is that Ap-V1 acquired during a larval tick bloodmeal may be later outcompeted by a subsequent infection of Ap-ha acquired during a nymphal bloodmeal. This phenomenon may be plausible because infection with either variant is maintained within the tick vector from one developmental stage to the next; higher prevalence of Ap-V1 in the nymphal stage did not yield a higher rate of Ap-V1 infection in adult ticks of the same cohort (*31*). Other possibilities include that *I. scapularis* larvae may be more likely to feed on deer than mice in certain geographic regions, that small-mammal populations in certain regions may not harbor Ap-ha, or that another small mammal may serve as an alternate reservoir for Ap-V1 in nature. The difference in Ap-ha prevalence between *I. scapularis* adults and nymphs could cause higher anaplasmosis incidence during the early spring and autumn months, when *I. scapularis* adults are most active, but investigating this possibility was beyond the scope of this study.

It is likely that competition between Ap-V1 and Ap-ha occurs primarily between particular Ap-V1 and Ap-ha clusters (Figures 4, 5). The region between these clusters should be a continued area of focus for epidemiologic research. Competition of variants at the local scale will likely result in spatial changes in the incidence of anaplasmosis; the expansion of Ap-ha clusters and large but unchanging clusters of Ap-V1 observed in our study match the dynamics of human anaplasmosis incidence depicted in Russell et al. (*13*) (Appendix Figure; Video). Our study showed tests for correlation using only Ap-ha in ERI calculations moderately increased the correlation between ERI and reported anaplasmosis incidence; this finding suggests that surveillance testing to detect *A. phagocytophilum* in host-seeking ticks must be variant-specific to yield the most accurate assessment of anaplasmosis risk.

**Video:** Anaplasmosis hotspots and coldspots and Bernoulli clusters of pathogenic and nonpathogenic genetic variants of *Anaplasma phagocytophilum* bacteria in adult and nymphal *Ixodes scapularis* ticks in New York, 2010–2018. Ap-ha, pathogenic variant; Ap-V1, nonpathogenic variant.

Our study had several limitations, including spatiotemporal variability in tick sampling and the limited spatial extent of our data. Collection sites are more numerous and in closer proximity to one another in the Capital District region than other regions (Figure 1). Heterogenous sampling effort likely resulted in larger clusters of Ap-V1 than Ap-ha (Table 2; Figures 4, 5), because the a priori parameters of the cluster analysis forced clusters to include the same maximum number of ticks, regardless of the distance between sites. Therefore, Ap-V1 clusters in the Western region required a larger radius to include the same number of ticks than Ap-ha clusters in the Capital District region, potentially limiting spatial resolution in western New York. Lower sample sizes at varying latitudes and longitudes also likely reduced statistical power of the ZINB models. In addition, the directionality of the emergence of new clusters and spatiotemporal interaction in the ZINB models are limited by the lack of data outside of New York, possibly biasing results.

Given the changes in spatial distribution of the Ap-ha variant of *A. phagocytophilum,* we suggest medical providers in newly emergent areas familiarize themselves with the signs and symptoms of anaplasmosis to streamline prompt and accurate diagnosis and treatment to ensure the best patient outcomes. Tickborne disease prevention education campaigns should target populations along the leading edge of Ap-ha advancement in New York and elsewhere. Continued differentiation and monitoring of the Ap-ha and Ap-V1 variants of *A. phagocytophilum* to document rate and directionality of spread is critical; further studies will elucidate the ecologic factors driving the expansion of Ap-ha and the resulting increase in anaplasmosis. The results of our study and others can be used to educate medical practitioners and to guide public health policy and disease prevention efforts in New York. 

Appendix Additional information about spatiotemporal associations of *Anaplasma phagocytophilum* bacteria variants in *Ixodes scapularis* ticks and humans, New York.
