# Supplementary figures and images for: Associations of Anaplasma phagocytophilum Bacteria Variants in Ixodes scapularis Ticks and Humans, New York, USA
Source: Emerg Infect Dis. 2023 Mar;29(3):540–50. doi: 10.3201/eid2903.220320 (PMC9973697; doi:10.3201/eid2903.220320)

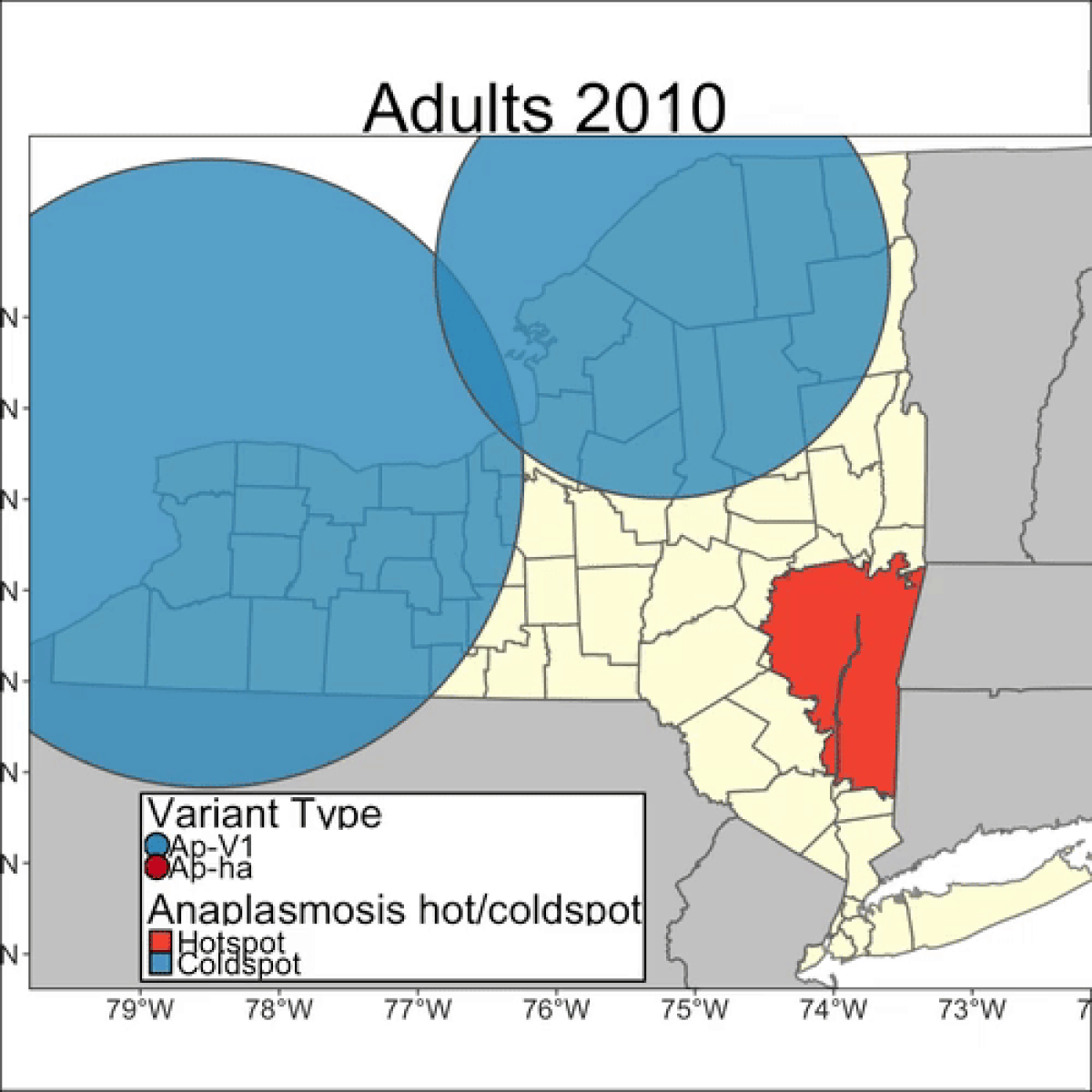

Supplement: Supplementary file 1 [file 22-0320-V.gif]
