# Supplementary material for: Associations of Anaplasma phagocytophilum Bacteria Variants in Ixodes scapularis Ticks and Humans, New York, USA
Source: Emerg Infect Dis. 2023 Mar;29(3):540–50. doi: 10.3201/eid2903.220320 (PMC9973697; doi:10.3201/eid2903.220320)
Supplement: Appendix — Additional information about spatiotemporal associations of Anaplasma phagocytophilum bacteria variants in Ixodes scapularis ticks and humans, New York. [file 22-0320-Techapp-s1.pdf]

Article DOI: https://doi.org/10.3201/eid2903.220320

Associations of *Anaplasma phagocytophilum* Bacteria Variants in *Ixodes scapularis* Ticks and Humans, New York, USA

Appendix

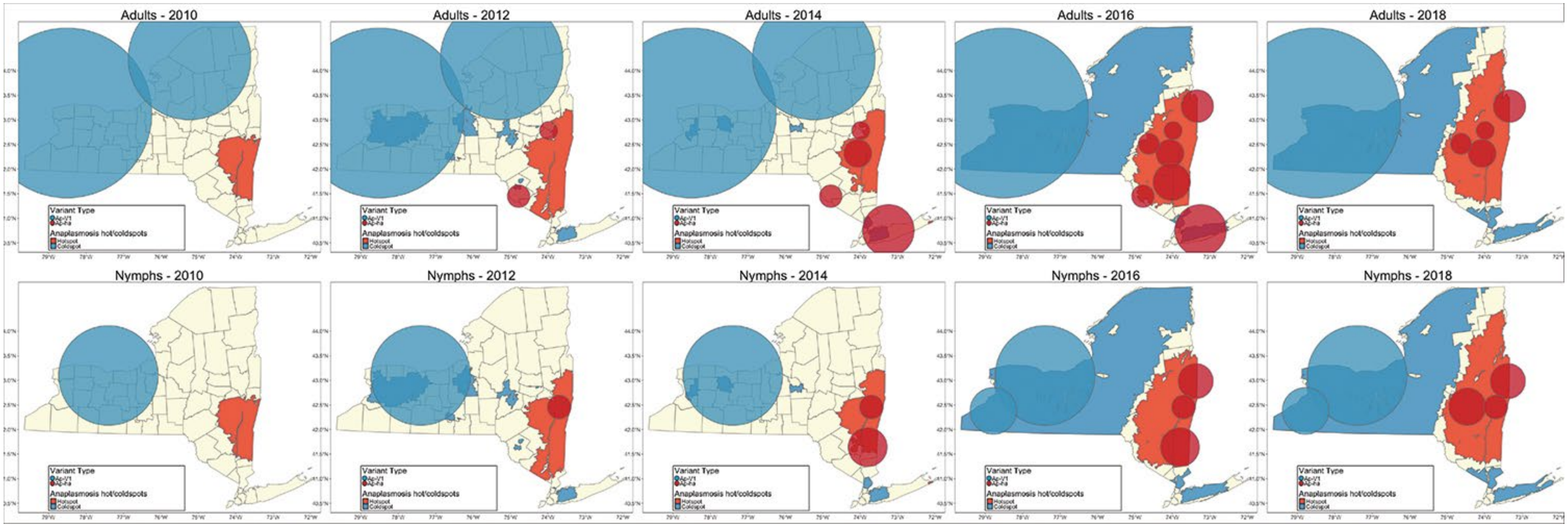

Appendix Figure. Anaplasmosis hot/coldspots (*1*) and Bernoulli clusters of pathogenic and nonpathogenic genetic variants of *Anaplasma phagocytophilum* bacteria in adult and nymph *Ixodes scapularis* ticks in New York, 2010, 2012, 2014, 2016, and 2018. Ap-ha, pathogenic variant; Ap-V1, nonpathogenic variant.

Reference

1. Russell A, Prusinski M, Sommer J, O'Connor C, White J, Falco R, et al. Epidemiology and spatial emergence of anaplasmosis, New York, USA, 2010–2018. Emerg Infect Dis. 2021;27:2154–62. PubMed https://doi.org/10.3201/eid2708.210133
